# Prasugrel Outperforms Ticagrelor in Acute Coronary Syndrome: A Real‐World Analysis

**DOI:** 10.1155/cdr/2457933

**Published:** 2026-10-17

**Authors:** Septi Melisa, Christianus Heru Setiawan, Chun-Yao Huang, Tian-Hsing Chen, Wei-Ting Chang, Wei-Pin Chang, Min-Huei Hsu, Christine Y. Lu, Jason C. Hsu

**Affiliations:** ^1^ International Ph.D. Program in Biotech and Healthcare Management College of Management Taipei Medical University Taipei Taiwan tmu.edu.tw; ^2^ School of Pharmacy Taipei Medical University Taipei Taiwan tmu.edu.tw; ^3^ Faculty of Pharmacy Sanata Dharma University Yogyakarta Indonesia usd.ac.id; ^4^ Division of Cardiology Taipei Medical University Hospital Taipei Taiwan tmuh.org.tw; ^5^ Department of Cardiology Chang Gung Memorial Hospital Keelung Taiwan cgmh.org.tw; ^6^ Division of Cardiovascular Medicine Chi Mei Hospital Tainan Taiwan; ^7^ School of Health Care Administration Taipei Medical University Taipei Taiwan tmu.edu.tw; ^8^ Clinical Data Center Office of Data Science Taipei Medical University Taipei Taiwan tmu.edu.tw; ^9^ Clinical Big Data Research Center Taipei Medical University Hospital Taipei Medical University Taipei Taiwan tmuh.org.tw; ^10^ Research Center of Data Science on Healthcare Industry College of Management Taipei Medical University Taipei Taiwan tmu.edu.tw; ^11^ Graduate Institute of Data Science College of Management Taipei Medical University Taipei Taiwan tmu.edu.tw; ^12^ Kolling Institute Faculty of Medicine and Health The University of Sydney and the Northern Sydney Local Health District Sydney New South Wales Australia; ^13^ School of Pharmacy Faculty of Medicine and Health The University of Sydney Sydney New South Wales Australia sydney.edu.au

**Keywords:** acute coronary syndrome, percutaneous coronary intervention, prasugrel, ticagrelor

## Abstract

Dual antiplatelet therapy (DAPT) with aspirin and a P2Y12 inhibitor is standard for patients with acute coronary syndrome (ACS) undergoing percutaneous coronary intervention (PCI), yet real‐world comparisons of ticagrelor and prasugrel remain limited. We evaluated their relative effectiveness and safety using the TriNetX Global Collaborative Network (2010–2024). Adult ACS patients receiving prasugrel or ticagrelor within 24 h post‐PCI were included. The primary endpoint was a 1‐year composite of all‐cause mortality, myocardial infarction (MI), and cerebral infarction; secondary endpoints included individual components, major bleeding, and repeat revascularization. Propensity score matching balanced baseline characteristics, with subgroup and sensitivity analyses assessing robustness. Among 14,895 matched pairs (*n* = 29,790) from 126,273 patients, prasugrel was associated with lower risk of the composite endpoint (HR 0.641; 95% CI 0.613–0.671), all‐cause mortality (HR 0.557; 95% CI 0.487–0.637), and MI (HR 0.648; 95% CI 0.616–0.680), without increased bleeding (HR 0.945; 95% CI 0.824–1.084). Repeat revascularization was higher with prasugrel (HR 1.791; 95% CI 1.639–1.957). Effects were consistent across subgroups. In this real‐world setting, prasugrel demonstrated superior effectiveness over ticagrelor in reducing mortality and ischemic events without increasing the risk of bleeding, although higher rates of repeat revascularization warrant further study. These findings support prasugrel as a potentially preferred P2Y12 inhibitor in selected ACS patients undergoing PCI.

## 1. Introduction

Dual antiplatelet therapy (DAPT), consisting of aspirin and a P2Y_12_ inhibitor, is a cornerstone treatment for patients with acute coronary syndrome (ACS) undergoing a percutaneous coronary intervention (PCI) to reduce the risk of restenosis and thrombotic events [1, 2]. However, while DAPT is effective in reducing thrombotic complications, it also increases the risk of bleeding, which has raised concerns regarding its overall clinical safety. To address this, several large‐scale randomized controlled trials were conducted to compare the efficacy and safety profiles of different P2Y_12_ inhibitors, including clopidogrel, prasugrel, and ticagrelor [3–7].

The 2025 guidelines for managing patients with ACS from the American College of Cardiology (ACC) and the American Heart Association (AHA) recommend prasugrel or ticagrelor over clopidogrel to reduce the risk of future stent thrombosis [8]. These guidelines are supported by findings from previous clinical trials, and by evidence that prasugrel and ticagrelor provide more rapid and consistent platelet inhibition than clopidogrel [4, 5, 9, 10]. However, a previous randomized controlled trial comparing ticagrelor and prasugrel found a significantly lower incidence of death, acute myocardial infarction, and stroke in the prasugrel group, with no difference in bleeding events between the two groups [5]. Although the results favor prasugrel over ticagrelor, there is ongoing debate regarding the integration of these findings into clinical guidelines [11, 12].

Despite insights from clinical trials, real‐world comparative data on ticagrelor and prasugrel remain limited [8, 13]. Therefore, in this study, we assessed and compared the safety and effectiveness of prasugrel and ticagrelor in patients with ACS undergoing a PCI procedure using real‐world data.

## 2. Methods

This retrospective cohort study adhered to the Strengthening the Reporting of Observational Studies in Epidemiology (STROBE) reporting guidelines (STROBE Checklist). Informed consent and review approval from the Taipei Medical University Institutional Review Board were not required for our study due to the Health Insurance Portability and Accountability Act (HIPAA) compliance of the database and the exclusive use of deidentified data.

### 2.1. Data Source and Study Population

This study used deidentified data from the TriNetX Global Collaborative Network, a network consisting of a total of 141 healthcare organizations (HCOs) [14]. Patients included in this study were diagnosed with ACS based on the International Classification of Diseases, Tenth Revision (ICD‐10) codes: I21, I22, I23, and I24.9. They underwent a PCI, identified using the Current Procedural Terminology (CPT), the ICD‐10 Procedure Coding System (ICD‐10‐PCS), and the Systematized Nomenclature of Medicine (SNOMED), within 1 month after the ACS diagnosis. The study period spanned January 1, 2010 (after the approval of prasugrel by US Food and Drug Administration), to December 31, 2024. The codes used to define PCI and outcomes are provided in Supporting Information (Tables S1 and S2).

The inclusion criteria included patients aged over 18 years who had received either prasugrel or ticagrelor within 1 day following the PCI procedure. To minimize outcome bias, we excluded patients with a history of prior bleeding events (gastrointestinal [GI] bleeding or hemorrhagic stroke) or liver dysfunction any number of days before treatment initiation, with no fixed lookback window, as defined by the diagnosis codes in Table S1. Additionally, patients using direct oral anticoagulants (dabigatran etexilate, rivaroxaban, apixaban, or edoxaban) were also excluded from the study.

### 2.2. Outcomes

The primary outcome of this study was a composite of all‐cause mortality, acute myocardial infarction, or cerebral infarction occurring between 1 day and 1 year following the index date, which was defined as the initiation of prasugrel or ticagrelor therapy after the PCI procedure. To ensure clinical relevance, only cases with a primary diagnosis of myocardial infarction were included, thereby excluding cases in which myocardial infarction was a secondary or incidental diagnosis rather than the primary reason for the visit. The secondary outcome focused on bleeding events, including GI bleeding and hemorrhagic stroke, to compare the safety profiles of each treatment cohort. Additionally, we evaluated individual components of primary composite outcome (all‐cause mortality, acute myocardial infarction, and cerebral infarction) as well as repeat revascularization as a separate secondary outcome. The specific diagnostic and procedural codes used to define these outcomes are provided in Table S2.

### 2.3. Subgroup Analysis

Subgroup analyses were conducted to examine whether the primary or secondary endpoints were influenced by patient characteristics, such as age, sex, or body mass index (BMI). Age was stratified at 65 years, consistent with its use as a threshold for elevated risk in prior antiplatelet therapy research [15]; sex was categorized as male and female. BMI was categorized as 18.5–25 kg/m^2^ and > 25 kg/m^2^ (the WHO threshold for overweight), used to stratify treatment effects rather than to define obesity. We also added subgroups based on certain comorbidities, such as Type 2 diabetes mellitus and kidney disease.

### 2.4. Sensitivity Analysis

A sensitivity analysis was performed to evaluate the robustness and consistency of the results while accounting for a potential immortal time bias across various follow‐up intervals ranging from 1 day after treatment initiation up to 5 years. Additionally, the cohort was stratified by ICD‐10 codes into patients diagnosed with ST‐elevation myocardial infarction (STEMI) and those with non–ST‐elevation myocardial infarction (NSTEMI).

### 2.5. Statistical Analysis

Baseline characteristics were summarized using the mean (standard deviation [SD]) for continuous variables and frequency (no. [%]) for categorical variables. A two‐tailed Z‐test was employed to compare baseline characteristics between the two groups for categorical variables, whereas a *t*‐test was utilized for continuous variables. To ensure balance in baseline covariates across cohorts, propensity score matching was performed prior to analysis using a greedy nearest neighbor approach with a 1:1 ratio without replacement and a caliper width of 0.1. The matching process included the following covariates: demographics, diagnoses, medications, and laboratory results (full list of covariates provided in Table S1). Balance between cohorts was assessed using the standardized mean difference (SMD), with an SMD of < 0.1 considered indicative of sufficient similarity and visualized through density plots (Figure S1). A survival analysis was conducted using the Kaplan–Meier method to estimate and compare the risk of outcomes between the two cohorts, with statistical significance evaluated using the log‐rank test. Hazard ratios (HRs) with corresponding 95% confidence intervals (CIs) were reported from a time‐to‐event analysis and two‐sided test with *p* < 0.05 considered significant. Cohort identification and data analysis were conducted using Query Builder and the analytic function in April to May 2025. The compare outcomes function within the analytic tool was utilized to perform time‐to‐event survival analyses and the log‐rank test. Cumulative incidence and a forest plot for subgroup analysis were created using Python vers. 3.12.7.

## 3. Results

Among the 126,273 patients aged at least 18 years old diagnosed with ACS who underwent a PCI within 6 months, 77,879 received ticagrelor and 14,899 received prasugrel within 1 day postprocedure, following application of specific exclusion criteria (Figure 1). Prior to matching, patients in the ticagrelor group were older (66.4 ± 11.6 vs. 64.3 ± 10.8 years, *p* < 0.001), had a higher proportion of females (27.43% vs. 21.96%, *p* < 0.001), and exhibited a lower average BMI (30.1 ± 6.4 vs. 30.8 ± 6.38 kg/m^2^, *p* < 0.001). Significant differences were observed in the history of acute kidney disease (8.238% vs. 6.16%) and neoplasms (7.50% vs. 5.82%, *p* < 0.001) within the ticagrelor group. Following propensity score matching, 14,895 patients were included in both the prasugrel and ticagrelor groups (Table 1).

**Figure 1:**
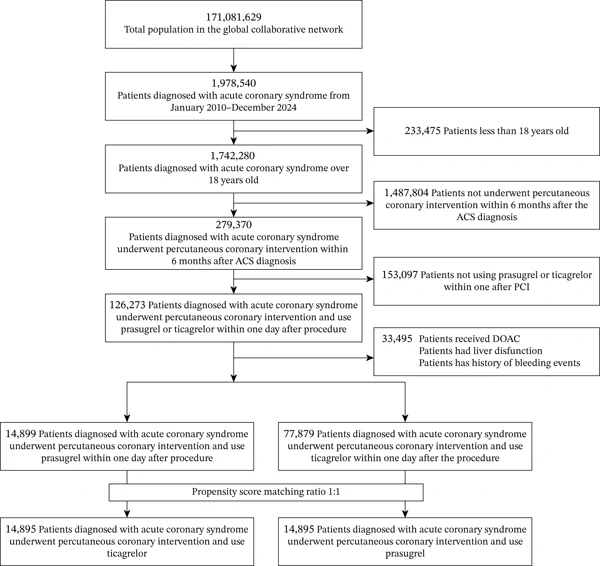
Patient selection flowchart.

**Table 1:** Baseline characteristics prior prasugrel or ticagrelor initiation before and after propensity score matching.

Characteristic name	Before matching				After matching			
	Prasugrel (*n* = 14,899)	Ticagrelor (*n* = 77,879)	*p*	SMD	Prasugrel (*n* = 14,895)	Ticagrelor (*n* = 14,895)	*p*	SMD
Current age, mean (SD)	64.33 (10.75)	66.39 (11.60)	< 0.001 ^∗^	0.184	64.34 (10.75)	64.85 (11.01)	< 0.001 ^∗^	0.047

Sex								
Male, *n* (%)	11206 (75.21%)	53029 (68.09%)	< 0.001 ^∗^	0.159	11202 (75.21%)	11273 (75.68%)	0.339	0.011
Female, *n* (%)	3272 (21.96%)	21363 (27.43%)	< 0.001 ^∗^	0.127	3272 (21.97%)	3219 (21.61%)	0.457	0.009

Ethnicity								
Not Hispanic or Latino	9772 (65.59%)	51555 (66.20%)	0.149	0.013	9769 (65.59%)	9914 (66.56%)	0.076	0.021
Hispanic or Latino	827 (5.55%)	4422 (5.68%)	0.538	0.006	826 (5.55%)	846 (5.68%)	0.615	0.006
Unknown Ethnicity	4300 (28.86%)	21902 (28.12%)	0.067	0.016	4300 (28.87%)	4135 (27.76%)	0.034 ^∗^	0.025

Race								
White, *n* (%)	9304 (62.45%)	52185 (67.01%)	< 0.001 ^∗^	0.096	9303 (62.46%)	10099 (67.80%)	< 0.001 ^∗^	0.112
Unknown race, *n* (%)	2706 (18.16%)	10113 (12.99%)	< 0.001 ^∗^	0.143	2705 (18.16%)	1823 (12.24%)	< 0.001 ^∗^	0.165
Black or African American	1153 (7.74%)	7809 (10.03%)	< 0.001 ^∗^	0.080	1153 (7.74%)	1413 (9.49%)	< 0.001 ^∗^	0.062
Asian	926 (6.22%)	4264 (5.48%)	< 0.001 ^∗^	0.032	925 (6.21%)	860 (5.77%)	0.113	0.018
Other race	499 (3.35%)	2382 (3.06%)	0.061	0.017	499 (3.35%)	498 (3.34%)	0.974	<0.001
BMI, mean (SD)	30.76 (6.38)	30.11 (6.40)	< 0.001 ^∗^	0.103	30.76 (6.38)	30.41 (6.34)	< 0.001 ^∗^	0.056
18.5–24.99 kg/m^2^	1091 (7.32%)	7076 (9.09%)	< 0.001 ^∗^	0.064	1091 (7.33%)	1114 (7.48%)	0.611	0.006
25–30 kg/m^2^	2606 (17.49%)	14276 (18.33%)	0.015 ^∗^	0.022	2606 (17.50%)	2598 (17.44%)	0.903	0.001
31–40 kg/m^2^	2398 (16.10%)	12427 (15.96%)	0.673	0.004	2398 (16.10%)	2398 (16.10%)	1	<0.001
40–100 kg/m^2^	622 (4.18%)	3025 (3.88%)	0.095	0.015	622 (4.18%)	551 (3.70%)	0.034 ^∗^	0.024

Disease history								
Acute myocardial infarction, *n* (%)	7466 (50.11%)	29743 (38.19%)	< 0.001 ^∗^	0.242	7462 (50.10%)	7558 (50.74%)	0.266	0.013
Chronic ischemic heart disease	6813 (45.73%)	29136 (37.41%)	< 0.001 ^∗^	0.169	6809 (45.71%)	6911 (46.40%)	0.236	0.014
Essential (primary) hypertension	5843 (39.22%)	29919 (38.42%)	0.066	0.016	5841 (39.22%)	6343 (42.59%)	< 0.001 ^∗^	0.069
Type 2 diabetes mellitus	3046 (20.44%)	16421 (21.09%)	0.078	0.016	3046 (20.45%)	2924 (19.63%)	0.077	0.020
Acute kidney failure and chronic kidney disease	1384 (9.29%)	9355 (12.01%)	< 0.001 ^∗^	0.088	1384 (9.29%)	1286 (8.63%)	0.047	0.023
Neoplasms	918 (6.16%)	6416 (8.24%)	< 0.001 ^∗^	0.080	918 (6.16%)	1147 (7.70%)	< 0.001 ^∗^	0.061
Hypertensive heart disease	747 (5.01%)	4808 (6.17%)	< 0.001 ^∗^	0.050	747 (5.02%)	1072 (7.20%)	< 0.001 ^∗^	0.091
Cerebral infarction	87 (0.58%)	1288 (1.65%)	< 0.001 ^∗^	0.102	87 (0.58%)	99 (0.67%)	0.377	0.010

Medications								
HMG CoA reductase inhibitors	6238 (41.87%)	25729 (33.04%)	< 0.001 ^∗^	0.183	6234 (41.85%)	6197 (41.61%)	0.664	0.005
Aspirin	5912 (39.68%)	24964 (32.06%)	< 0.001 ^∗^	0.160	5908 (39.66%)	5917 (39.73%)	0.915	0.001
Beta blocking agents	5137 (34.48%)	22097 (28.37%)	< 0.001 ^∗^	0.132	5134 (34.47%)	5083 (34.13%)	0.534	0.007
Vasodilators	5007 (33.61%)	20092 (25.80%)	< 0.001 ^∗^	0.171	5004 (33.60%)	4938 (33.15%)	0.417	0.009
Agents acting on the renin‐angiotensin system	3627 (24.34%)	16864 (21.65%)	< 0.001 ^∗^	0.064	3624 (24.33%)	3518 (23.62%)	0.150	0.017
Antiarrhythmics	3165 (21.24%)	15322 (19.67%)	< 0.001 ^∗^	0.039	3162 (21.23%)	3194 (21.44%)	0.651	0.005
ACE inhibitors	2340 (15.71%)	9904 (12.72%)	< 0.001 ^∗^	0.086	2338 (15.70%)	2145 (14.40%)	0.002 ^∗^	0.036
Calcium channel blockers	2316 (15.55%)	11381 (14.61%)	0.003 ^∗^	0.026	2316 (15.55%)	2260 (15.17%)	0.368	0.010
Diuretics	2133 (14.32%)	11811 (15.17%)	0.008 ^∗^	0.024	2132 (14.31%)	2272 (15.25%)	0.022 ^∗^	0.026
Insulins and analogues	2107 (14.14%)	10779 (13.84%)	0.330	0.009	2106 (14.14%)	2030 (13.63%)	0.203	0.015
Angiotensin II receptors blockers (ARBs)	1418 (9.52%)	7628 (9.80%)	0.296	0.009	1417 (9.51%)	1530 (10.27%)	0.028 ^∗^	0.025
Osmotically acting laxatives	1511 (10.14%)	7926 (10.18%)	0.895	0.001	1510 (10.14%)	1627 (10.92%)	0.027 ^∗^	0.026
Softeners, emollients	1209 (8.12%)	6810 (8.74%)	0.012 ^∗^	0.023	1208 (8.11%)	1388 (9.32%)	< 0.001 ^∗^	0.043
metformin	1061 (7.12%)	4859 (6.24%)	< 0.001 ^∗^	0.035	1061 (7.12%)	902 (6.06%)	< 0.001 ^∗^	0.043
Sulfonylureas	440 (2.95%)	2347 (3.01%)	0.692	0.004	440 (2.95%)	424 (2.85%)	0.581	0.006
Glucagon‐like peptide‐1 (GLP‐1) analogues	251 (1.69%)	1204 (1.55%)	0.212	0.011	251 (1.69%)	220 (1.48%)	0.150	0.017
Dipeptidyl peptidase 4 (DPP‐4) inhibitors	215 (1.44%)	1130 (1.45%)	0.941	0.001	215 (1.44%)	196 (1.32%)	0.345	0.011
Sodium‐glucose cotransporter 2 (SGLT2) inhibitors	293 (1.97%)	1480 (1.90%)	0.589	0.005	293 (1.97%)	277 (1.86%)	0.499	0.008
Empagliflozin	185 (1.24%)	946 (1.22%)	0.783	0.002	185 (1.24%)	164 (1.10%)	0.258	0.013
Dapagliflozin	86 (0.58%)	484 (0.62%)	0.526	0.006	86 (0.58%)	99 (0.67%)	0.338	0.011
Alpha glucosidase inhibitors	14 (0.09%)	44 (0.06%)	0.094	0.014	14 (0.09%)	10 (0.07%)	0.414	0.009

Laboratory results								
Platelets [#/volume] in blood, mean (SD)	237.36 (73.57)	238.04 (77.38)	0.454	0.009	237.35 (73.59)	238.00 (74.09)	0.560	0.009
0–150000 10 ^∗^3/uL, *n* (%)	8640 (57.99%)	38453 (49.38%)	< 0.001 ^∗^	0.173	8636 (57.98%)	8720 (58.54%)	0.324	0.011
150001–450000 10 ^∗^3/uL, *n* (%)	10 (0.07%)	117 (0.15%)	0.012 ^∗^	0.025	10 (0.07%)	18 (0.12%)	0.130	0.018
450001–1000000 10 ^∗^3/uL, *n* (%)	10 (0.07%)	10 (0.01%)	< 0.001 ^∗^	0.027	10 (0.07%)	0 (0.00%)	0.002 ^∗^	0.037
Hemoglobin [mass/volume] in blood	13.99 (2.04)	13.62 (2.25)	< 0.001 ^∗^	0.172	13.99 (2.04)	13.99 (2.07)	0.816	0.004
0–10 g/dL, *n* (%)	598 (4.01%)	3971 (5.10%)	< 0.001 ^∗^	0.052	598 (4.02%)	517 (3.47%)	0.013 ^∗^	0.029
10–1000 g/dL, *n* (%)	8357 (56.09%)	37665 (48.36%)	< 0.001 ^∗^	0.155	8355 (56.09%)	8534 (57.29%)	0.036 ^∗^	0.024
Creatinine [mass/volume] in serum, plasma or blood, mean (SD)	1.37 (5.40)	1.65 (7.33)	0.001 ^∗^	0.044	1.37 (5.40)	1.52 (7.09)	0.119	0.024
Leukocytes [#/volume] in blood	14.48 (151.29)	12.96 (131.99)	0.374	0.011	14.48 (151.33)	11.10 (94.90)	0.092	0.027
Neutrophils [#/volume] in blood	43.77 (518.13)	45.62 (518.19)	0.789	0.004	43.80 (518.28)	36.49 (491.31)	0.392	0.014
Albumin [mass/volume] in serum, plasma or blood	3.98 (0.50)	3.96 (0.54)	0.003 ^∗^	0.042	3.98 (0.50)	4.00 (0.51)	0.002 ^∗^	0.053
INR in plasma or blood	1.07 (0.28)	1.08 (0.27)	0.002 ^∗^	0.045	1.07 (0.28)	1.06 (0.21)	0.185	0.025
0.8–1.5 {INR}	5376 (36.08%)	21573 (27.70%)	< 0.001 ^∗^	0.181	5372 (36.07%)	5411 (36.33%)	0.638	0.005
1.5–200000 {INR}	203 (1.36%)	1067 (1.37%)	0.942	0.001	203 (1.36%)	165 (1.11%)	0.046 ^∗^	0.023
Cholesterol in HDL [mass/volume] in serum or plasma	41.90 (14.20)	41.65 (16.85)	0.344	0.016	41.90 (14.20)	41.05 (16.15)	0.006 ^∗^	0.055
Triglyceride [mass/volume] in serum, plasma or blood	170 (15444.67%)	166.21 (160.98)	0.108	0.026	170.24 (154.47)	167.64 (166.15)	0.420	0.016
Cholesterol in LDL [mass/volume] in serum or plasma	109 (4310.58%)	106.87 (44.35)	< 0.001 ^∗^	0.059	109.45 (43.11)	109.02 (44.31)	0.626	0.010
Hemoglobin A1c/hemoglobin.total in blood	7 (199.36%)	6.95 (2.01)	0.482	0.012	6.92 (1.99)	6.79 (1.96)	0.003 ^∗^	0.066
Troponin I. cardiac [Mass/volume] in serum, plasma or blood	4 (1320.16%)	3.48 (16.65)	0.100	0.032	3.97 (13.21)	3.60 (13.94)	0.259	0.027
0–10 ng/mL	3578 (24.02%)	12180 (15.64%)	< 0.001 ^∗^	0.211	3575 (24.00%)	2983 (20.03%)	< 0.001 ^∗^	0.096
10–10000 ng/mL	696 (4.67%)	1732 (2.22%)	< 0.001 ^∗^	0.134	696 (4.67%)	453 (3.04%)	< 0.001 ^∗^	0.085
Corrected QT interval (QTc)	433.68 (33.09)	434.20 (34.00)	0.608	0.016	433.68 (33.09)	431.31 (33.10)	0.042 ^∗^	0.072
Left ventricular ejection fraction (LVEF) (%)	51.39 (14.13)	52.41 (14.00)	0.142	0.073	51.39 (14.13)	52.22 (13.66)	0.326	0.060

Abbreviations: ACE, angiotensin converting enzymes; DPP‐4, dipeptidyl peptidase 4; GLP‐1, glucagon‐like peptide‐1; HDL, high density lipoprotein; INR, international normalized ratio; LDL, low density lipoprotein; SD, standard deviation; SGLT‐2, sodium‐glucose cotransporter‐2; SMD, standard mean difference.

^∗^ *p* < 0.05.

At 1‐year post initiation, 3073 patients (20.631%) in the prasugrel group had experienced the primary endpoints of all‐cause mortality, a primary diagnosis of myocardial infarction, and cerebral infarction, compared with 4513 patients (30.299%) in the ticagrelor group (HR 0.641 [95% CI 0.613–0.671]; log‐rank *p* < 0.0001, Figure 2A). However, there was no significant difference in bleeding events between the two groups (HR 0.945 [95% CI 0.824–1.084]; log‐rank *p* < 0.4228) (Figure 3). The Kaplan–Meier curve for bleeding events is shown in Figure 2B. Similarly, patients receiving prasugrel were less likely to experience all‐cause mortality than those receiving ticagrelor (HR 0.6 [95% CI 0.4–0.8]; log‐rank *p* < 0.001, Figure 2C). Patients receiving prasugrel were also less likely to experience acute myocardial infarction (HR 0.647 [95% CI 0.616–0.680]; log‐rank *p* < 0.001, Figure 2D) and cerebral infarction (HR 0.701 [95% CI 0.583–0.843]; log‐rank *p* < 0.0001, Figure 2E) than those receiving ticagrelor. Interestingly, repeat revascularization was more common in the prasugrel group (9.1%) than in the ticagrelor group (5.1%) (HR 1.791 [95% CI: 1.639–1.957]; log‐rank *p* < 0.0001, Figure 2F), suggesting that ticagrelor may offer greater protection against the need for subsequent procedures.

**Figure 2:**
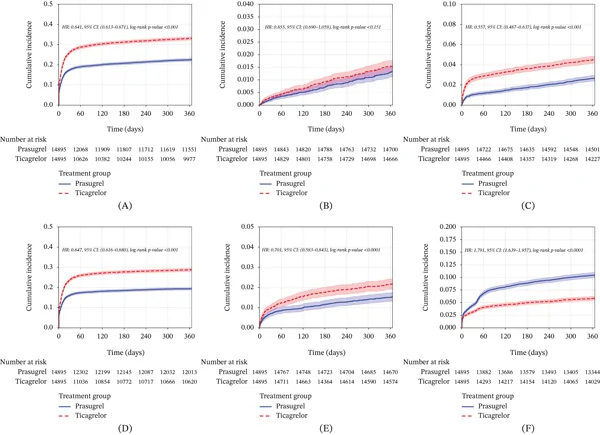
Cumulative Incidence for all outcomes 1 year after treatment initiation. This figure presents cumulative incidence curves for time‐to‐event analyses of six outcomes: (A) primary endpoints, (B) bleeding events, (C) all‐cause mortality, (D) acute myocardial infarction, (E) cerebral infarction, and (F) repeat revascularization. The number‐at‐risk tables below each plot detail participant retention across the follow‐up period.

**Figure 3:**
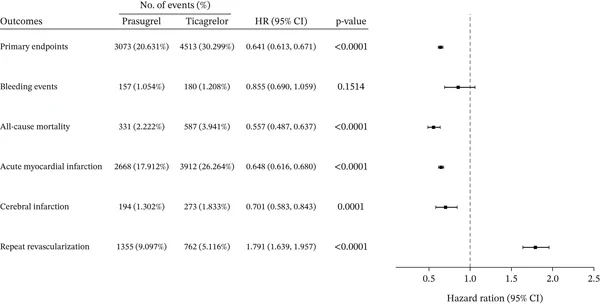
Clinical outcome comparison between prasugrel and ticagrelor. The outcomes analyzed include the composite primary endpoint, bleeding events, all‐cause mortality, acute myocardial infarction, cerebral infarction, and repeat revascularization. Hazard ratios less than 1 indicate a lower risk with prasugrel, whereas values greater than 1 indicate a higher risk. Notably, prasugrel was associated with significantly lower risks of the primary endpoint, all‐cause mortality, myocardial infarction, and cerebral infarction, but a higher risk of repeat revascularization compared with ticagrelor.

Subgroup analyses revealed that prasugrel was consistently associated with a reduced risk of primary endpoints compared with ticagrelor across all examined subgroups. The overall HR favored prasugrel (HR: 0.681; 95% CI: 0.616–0.752), with similar benefits observed in both males (HR: 0.594; 95% CI: 0.523–0.673) and females (HR: 0.687; 95% CI: 0.558–0.847), as well as in patients aged 18–65 years (HR: 0.663; 95% CI: 0.553–0.796) and those ≥ 66 years (HR: 0.682; 95% CI: 0.603–0.772). Favorable outcomes for prasugrel also extended to BMI categories, including those with a BMI of 18.5–25 kg/m^2^ (HR: 0.696; 95% CI: 0.546–0.887) and a BMI of > 25 kg/m^2^ (HR: 0.683; 95% CI: 0.594–0.785). No statistically significant difference was found for bleeding events between the two treatments in the overall population (HR: 0.953; 95% CI: 0.830–1.095). No statistically significant difference was detected across sex‐ and BMI‐based subgroups, although a modest trend toward a lower bleeding risk with prasugrel was noted in patients aged ≥ 66 years (HR: 0.829; 95% CI: 0.689–0.998). Prasugrel was also associated with a significantly lower risk of all‐cause mortality (HR: 0.941; 95% CI: 0.915–0.968), with consistent benefits across sex, BMI, and comorbidity strata. In contrast, for cerebral infarction, no statistically difference was detected between prasugrel and ticagrelor in female patients and those with kidney disease (see Figure S2). However, given the smaller subgroup sizes and lack of adjustment for multiple comparisons, these findings should not be interpreted as evidence of equivalent risk.

Figure S3 and Table S3 illustrate sensitivity analyses comparing prasugrel and ticagrelor across a range of cardiovascular outcomes over various follow‐up periods. Across follow‐up windows, prasugrel consistently showed a reduced risk of primary endpoint, all‐cause mortality, and myocardial infarction compared with ticagrelor, especially during short‐term follow‐up. For myocardial infarction, all time frames consistently demonstrated a clear advantage for prasugrel. In contrast, bleeding events revealed mixed results; most comparisons showed no significant difference, suggesting similar safety profiles in terms of bleeding risk. Cerebral infarction outcomes also leaned in favor of prasugrel, with a significantly reduced risk in early follow‐up, although some longer‐term follow‐up periods showed nonsignificant results, indicating reduced certainty. Repeat revascularization consistently favored ticagrelor across all time windows (e.g., HR: 2.129; CI: 1.881–2.410 for 1 month–1 year). Overall, prasugrel appeared superior in reducing ischemic events and mortality, whereas ticagrelor may be more effective in preventing repeat interventions.

When stratified by STEMI and NSTEMI, the choice of the follow‐up window could influence estimated risk differences between prasugrel and ticagrelor. For instance, certain intervals showed no significant difference in all‐cause mortality between the two groups, whereas longer follow‐up (up to 3 or 5 years) favored prasugrel for bleeding events in STEMI patients (see Figures S4 and S5).

## 4. Discussion

In this retrospective study, the safety and efficacy of prasugrel versus ticagrelor were assessed in patients with ACS who underwent a PCI, utilizing deidentified data from a global collaborative network. The findings suggested that patients receiving prasugrel exhibited a lower likelihood of experiencing primary endpoints, which were all‐cause mortality, acute myocardial infarction, and cerebral infarction, compared with those receiving ticagrelor. However, with respect to the risk of bleeding, no significant difference was observed between the two groups. It was also found that prasugrel was associated with a higher risk of repeat revascularization compared with ticagrelor.

The findings of this study aligned with those of the ISAR‐REACT 5 trial, a pivotal randomized controlled study comparing prasugrel and ticagrelor in patients with ACS. In ISAR‐REACT 5, prasugrel was associated with a significantly lower incidence of the composite endpoint of death, myocardial infarction, or stroke at 1‐year follow‐up, compared with ticagrelor [5]. This result was somewhat unexpected, as ISAR‐REACT 5 was the first head‐to‐head randomized trial to demonstrate superiority of prasugrel over ticagrelor for the composite endpoint of death, myocardial infarction, or stroke, challenging prior expectations based on earlier studies that had suggested comparable efficacy between the two agents [16]. An observational study utilizing a German statutory health insurance claims database found similar results [17]. In contrast, the PRAGUE‐18 trial, a head‐to‐head randomized comparison of prasugrel and ticagrelor in STEMI patients undergoing a primary PCI, reported no significant difference between the two agents in the primary composite endpoint of death, reinfarction, urgent target vessel revascularization, stroke, or serious bleeding requiring transfusion or hospitalization within 7 days [6]. This result remained consistent even after a 1‐year follow‐up, although it may have been influenced by a high rate of switching to clopidogrel, potentially driven by reimbursement issues [18]. The TRILOGY ACS trial found no significant benefit of prasugrel over clopidogrel in medically managed ACS patients over 30 months of follow‐up [19]. This suggests that prasugrel′s benefit may be greater in patients undergoing invasive PCI‐based strategies than in conservatively managed populations.

In contrast, a meta‐analysis of four randomized controlled trials of prasugrel and ticagrelor comparisons found no significant difference in terms of mortality, myocardial infarction, MACE, stroke, or stent thrombosis [20]. However, the component trials in this meta‐analysis were generally small, and PRAGUE‐18 was the only trial specifically designed and powered to assess clinical endpoints; these findings should therefore be interpreted with caution. This pooled analysis highlighted the complexity and inconsistency of existing evidence regarding the comparative effectiveness of these two potent P2Y_12_ inhibitors. Similarly, a Swedish registry found no significant differences in major adverse cardiovascular and cerebrovascular events (MACCEs) or major bleeding between ticagrelor and prasugrel in routine clinical practice [21]. However, results from another trial suggested that preprocedural treatment with ticagrelor was linked to a higher coronary flow (Q) and lower microcirculatory resistance (R) compared with prasugrel, indicating that ticagrelor may enhance postprocedural coronary flow and improve microvascular function [22]. Furthermore, conflicting evidence exists regarding the effect of ticagrelor on microvascular function. One study examining coronary physiology post‐PCI demonstrated that ticagrelor was not superior to prasugrel in reducing the index of microcirculatory resistance (IMR), a marker of microvascular dysfunction [22]. Collectively, these discrepancies underscore the heterogeneity of study populations, methodologies, and clinical endpoints across trials and observational cohorts.

Our study yielded an intriguing finding regarding repeat revascularization. Despite prasugrel′s superior efficacy in preventing ischemic events, its use was associated with a higher risk of repeat revascularization, an observation that may appear paradoxical. This result aligns with a previous study that reported lower rates of target vessel revascularization in the ticagrelor group [23]. However, the TriNetX platform lacks the ability to distinguish between urgent and staged PCI procedures or to identify the specific vessel involved, which may limit the granularity of the interpretation. The exact cause of the increased revascularization rate among prasugrel users remains uncertain but may be influenced by the procedural complexity or baseline anatomical disease severity, factors not captured in the dataset. Therefore, this finding likely reflects underlying differences in patient characteristics and unmeasured confounding, rather than a direct pharmacologic consequence of prasugrel use.

The differing pharmacodynamic profiles of prasugrel and ticagrelor may contribute to the observed lower risk of primary endpoints in patients treated with prasugrel. Prasugrel is a third‐generation thienopyridine that undergoes rapid hepatic conversion to an active metabolite, which binds irreversibly to the platelet P2Y_12_ receptor [24–26]. This permanent blockade inhibits adenosine diphosphate (ADP)‐induced platelet activation and aggregation throughout the lifespan of the platelet. In contrast, ticagrelor belongs to the cyclopentyl‐triazolopyrimidine class of antiplatelets that do not require metabolic activation and exerts its effect by reversibly binding to the P2Y_12_ receptor [27–29]. This pharmacological distinction may partially explain the differences in clinical outcomes, including the incidence of stent thrombosis and major adverse cardiovascular events. Additionally, ticagrelor was shown to interact pharmacodynamically with aspirin, particularly at higher aspirin doses, which may attenuate its clinical benefits. This interaction has not been observed with prasugrel, suggesting a more‐favorable compatibility profile when combined with standard‐dose aspirin [16, 30].

To our knowledge, this is the first observational study comparing prasugrel and ticagrelor using real‐world data from a global collaborative network database. Despite the strengths of this study, including the use of a large, real‐world dataset derived from a global collaborative network that enhances the generalizability of the findings, several limitations should be acknowledged. As an observational study, it is inherently susceptible to unmeasured confounding factors that may have influenced the observed outcomes. Although we employed rigorous methods to minimize bias, it is possible that variables not captured in the dataset, such as medication adherence, socioeconomic factors, or specific clinical decision‐making processes, could have affected the results. An additional limitation is that prasugrel′s once‐daily dosing regimen, compared with ticagrelor′s twice‐daily regimen, may result in differential real‐world medication adherence between the two groups, potentially biasing outcomes in favor of prasugrel; adherence data were not available within TriNetX to evaluate this directly. Additionally, the retrospective nature of the analysis limited our ability to establish causality. Second, our study did not capture the exact duration of medication use, particularly with regard to DAPT, including aspirin and P2Y_12_ inhibitors. The duration of DAPT is a critical factor that may influence clinical outcomes, such as ischemic and bleeding events [31, 32]. To mitigate this potential source of bias, we included aspirin use as a covariate in the matching process, aiming to account for differences in antiplatelet therapy exposure. Third, our study did not specify the precise dosages of prasugrel and ticagrelor administered on the index date. According to guidelines, the recommended dosages for prasugrel vary across different regions. By not specifying these dosages, we aim to include a broader range of patients in our study. Fourth, the platform was unable to differentiate between outcomes originating from outpatient or inpatient settings, resulting in a significant risk of overestimating myocardial infarction occurrences and consequently leading to a notably high rate of hospitalizations. To mitigate this risk, we included only primary diagnoses, which typically indicate higher clinical urgency and suggest an acute or critical condition. Additionally, subgroup analyses were not adjusted for multiple comparisons, and some subgroups (e.g., women, patients with kidney disease) may have been underpowered to detect true differences between treatments; findings in these subgroups, including CIs crossing unity, should not be interpreted as evidence of equivalent risk. Despite these limitations, the study provides valuable insights into real‐world clinical practice, where randomized controlled trial conditions are often not fully replicated, and helps bridge the gap between trial‐based evidence and routine patient care.

## 5. Conclusions

Prasugrel was associated with a significantly lower risk of primary endpoints compared with ticagrelor, without a significant difference in bleeding risk. This suggests that the clinical benefit of prasugrel may not come at the cost of increased bleeding, an important consideration when selecting antiplatelet therapy in patients at elevated bleeding risk.

**Nomenclature**
ACC: American College of Cardiology
ACS: acute coronary syndrome
ADP: adenosine diphosphate
AHA: American Heart Association
BMI: body‐mass index
CI: confidence interval
DAPT: dual antiplatelet
GI: gastrointestinal
HCO: healthcare organization
HR: hazard ratio
ICD: International Classification of Diseases
IMR: index of microcirculatory resistance
MACCE: major adverse cardiovascular and cerebrovascular event
MACE: major adverse cardiovascular event
NSTEMI: non–ST‐elevation myocardial infarction
PCI: percutaneous coronary intervention
SMD: standardized mean difference
SNOMED: Systematized Nomenclature of Medicine
STEMI: ST‐elevation myocardial infarction
STROBE: Strengthening the Reporting of Observational Studies in Epidemiology

## Author Contributions

Concept and design: S.M., C.H.S., and J.C.H. Acquisition, analysis, or interpretation of data: S.M., C.H.S., C‐Y.H., T‐H.C., W‐T.C., W‐P.C., M‐H.H., C.Y.L., and J.C.H. Drafting of the manuscript: S.M. Critical review of the manuscript for important intellectual content: S.M., C.H.S., C‐Y.H., T‐H.C., W‐T.C., W‐P.C., M‐H.H., C.Y.L., and J.C.H. Statistical analysis: S.M. Obtained funding: J.C.H. Administrative, technical, or material support: S.M. and J.C.H. Supervision: J.C.H.

## Funding

This study was supported by the National Science and Technology Council (10.13039/100020595; NSTC113‐2634‐F038‐001, 114‐2628‐B‐038‐002‐MY3) and the Ministry of Education (10.13039/100010002; DP2‐TMU‐114‐A‐01).

## Conflicts of Interest

The authors declare no conflicts of interest.

## Supporting information


**Supporting Information** Additional supporting information can be found online in the Supporting Information section. STROBE Checklist. Table S1: Covariable definition. Table S2: Outcome definition. Table S3: Sensitivity analysis by outcome and follow‐up time. Figure S1: Distribution of propensity scores before and after matching. Figure S2: Subgroup analysis. Figure S3: Forest plot for sensitivity analysis of patient with acute coronary syndrome. Figure S4: Forest plot for sensitivity analysis of patient with STEMI. Figure S5: Forest plot for sensitivity analysis of patient with NSTEMI.

## Data Availability

Data sharing is not applicable to this article as no datasets were generated or analyzed during the current study.
