# Supplementary material for: Prasugrel Outperforms Ticagrelor in Acute Coronary Syndrome: A Real‐World Analysis
Source: Cardiovasc Ther. 2026 Oct 17;2026:2457933. doi: 10.1155/cdr/2457933 (PMC13675845; doi:10.1155/cdr/2457933)
Supplement: Supplementary file 1 [file CDR-2026-2457933-s001.docx]

**Supplementary Material**

Supplementary STROBE Checklist

Supplementary Table S1 Covariable Definition

Supplementary Table S2 Outcome Definition

Supplementary Table S3 Sensitivity Analysis by Outcome and Follow-Up Time

Supplementary Figure S1 Distribution of propensity scores before and after matching

Supplementary Figure S2 Subgroup Analysis

Supplementary Figure S3 Forest Plot for Sensitivity Analysis of Patient with Acute Coronary Syndrome

Supplementary Figure S4 Forest Plot for Sensitivity Analysis of Patient with STEMI

Supplementary Figure S5 Forest Plot for Sensitivity Analysis of Patient with NSTEMI

This supplemental material has been provided by the authors to give readers additional

information about their work.

**Supplementary STROBE Checklist.**

**STROBE Statement—Checklist of items that should be included in reports of *cohort studies***

|  | Item No | Recommendation | Page No |
| --- | --- | --- | --- |
| **Title and abstract** | 1 | (*a*) Indicate the study’s design with a commonly used term in the title or the abstract | 1,2 |
|  |  | (*b*) Provide in the abstract an informative and balanced summary of what was done and what was found | 2 |
| Introduction | | | |
| Background/rationale | 2 | Explain the scientific background and rationale for the investigation being reported | 3 |
| Objectives | 3 | State specific objectives, including any prespecified hypotheses | 3 |
| Methods | | | |
| Study design | 4 | Present key elements of study design early in the paper | 4 |
| Setting | 5 | Describe the setting, locations, and relevant dates, including periods of recruitment, exposure, follow-up, and data collection | 4 |
| Participants | 6 | (*a*) Give the eligibility criteria, and the sources and methods of selection of participants. Describe methods of follow-up | 4 |
|  |  | (*b*) For matched studies, give matching criteria and number of exposed and unexposed |  |
| Variables | 7 | Clearly define all outcomes, exposures, predictors, potential confounders, and effect modifiers. Give diagnostic criteria, if applicable | 4,5 |
| Data sources/ measurement | 8* | For each variable of interest, give sources of data and details of methods of assessment (measurement). Describe comparability of assessment methods if there is more than one group | 4,6 |
| Bias | 9 | Describe any efforts to address potential sources of bias | 6 |
| Study size | 10 | Explain how the study size was arrived at | 5 |
| Quantitative variables | 11 | Explain how quantitative variables were handled in the analyses. If applicable, describe which groupings were chosen and why | 9 |
| Statistical methods | 12 | (*a*) Describe all statistical methods, including those used to control for confounding | 6 |
|  |  | (*b*) Describe any methods used to examine subgroups and interactions | 5 |
|  |  | (*c*) Explain how missing data were addressed |  |
|  |  | (*d*) If applicable, explain how loss to follow-up was addressed |  |
|  |  | (*e*) Describe any sensitivity analyses | 5 |
| Results | | |  |
| Participants | 13* | (a) Report numbers of individuals at each stage of study—eg numbers potentially eligible, examined for eligibility, confirmed eligible, included in the study, completing follow-up, and analysed | 7 |
|  |  | (b) Give reasons for non-participation at each stage | 7 |
|  |  | (c) Consider use of a flow diagram | 7 |
| Descriptive data | 14* | (a) Give characteristics of study participants (eg demographic, clinical, social) and information on exposures and potential confounders | 7 |
|  |  | (b) Indicate number of participants with missing data for each variable of interest | 7 |
|  |  | (c) Summarise follow-up time (eg, average and total amount) | 7 |
| Outcome data | 15* | Report numbers of outcome events or summary measures over time | 7 |

| Main results | 16 | (*a*) Give unadjusted estimates and, if applicable, confounder-adjusted estimates and their precision (eg, 95% confidence interval). Make clear which confounders were adjusted for and why they were included | 7 |
| --- | --- | --- | --- |
|  |  | (*b*) Report category boundaries when continuous variables were categorized | 7 |
|  |  | (*c*) If relevant, consider translating estimates of relative risk into absolute risk for a meaningful time period | 7 |
| Other analyses | 17 | Report other analyses done—eg analyses of subgroups and interactions, and sensitivity analyses | 8 |
| Discussion | | | |
| Key results | 18 | Summarise key results with reference to study objectives | 9 |
| Limitations | 19 | Discuss limitations of the study, taking into account sources of potential bias or imprecision. Discuss both direction and magnitude of any potential bias | 13 |
| Interpretation | 20 | Give a cautious overall interpretation of results considering objectives, limitations, multiplicity of analyses, results from similar studies, and other relevant evidence | 13 |
| Generalisability | 21 | Discuss the generalisability (external validity) of the study results | 13 |
| Other information | | | |
| Funding | 22 | Give the source of funding and the role of the funders for the present study and, if applicable, for the original study on which the present article is based | 16 |

*Give information separately for exposed and unexposed groups.

**Note:** An Explanation and Elaboration article discusses each checklist item and gives methodological background and published examples of transparent reporting. The STROBE checklist is best used in conjunction with this article (freely available on the Web sites of PLoS Medicine at http://www.plosmedicine.org/, Annals of Internal Medicine at http://www.annals.org/, and Epidemiology at http://www.epidem.com/). Information on the STROBE Initiative is available at http://www.strobe-statement.org.

**Supplementary Table S1 Covariable Definition**

| **Covariable** | **Definition** |
| --- | --- |
| **Demographic** |  |
| Age | TrinetX demographic variable: Current age |
| Gender | TrinetX demographic variable: Sex |
| Ethnicity | TrinetX demographic variable: Ethnicity |
| Race | TrinetX demographic variable: Race |
| BMI | TNX curated 9083 |
| **Diagnoses** |  |
| Acute Myocardial Infarction | ICD-10-CM I21 |
| Chronic Ischemic heart disease | ICD-10-CM I25 |
| Essential (primary) hypertension | ICD-10-CM I10 |
| Hypertensive heart disease | ICD-10-CM I11 |
| Cerebral Infarction | ICD-10-CM I63 |
| Type 2 Diabetes Mellitus | ICD-10-CM E11 |
| Neoplasm | ICD-10-CM C00 - D49 |
| Acute Kidney Failure and Chronic Kidney Disease | ICD-10-CM N17 – N19 |
| **Medications** |  |
| Aspirin | RxNorm 1191 |
| Beta blocking agents | ATC C07A |
| Agents acting on the renin-angiotensin system | ATC C09 |
| Calcium channel blockers | ATC C08 |
| Vasodilators used in cardiac diseases | ATC C01D |
| Ace inhibitors, plain | ATC C09A |
| Angiotensin ii receptor blockers (ARBs, plain) | ATC C09C |
| Hmg coa reductase inhibitors (Statins) | ATC C10AA |
| Metformin | RxNorm 6809 |
| Sodium-glucose co-transporter 2 (SGLT2) inhibitors | ATC A10BK |
| Empagliflozin | RxNorm 1545653 |
| Dapagliflozin | RxNorm 1488564 |
| Alpha glucosidase inhibitors | ATC A10BF |
| Sulfonylureas | ATC A10BB |
| Dipeptidyl peptidase 4 (dpp-4) inhibitors | ATC A10BH |
| Glucagon-like peptide-1 (glp-1) analogues | ATC A10BJ |
| **Laboratorium Result** |  |
| INR | TNX curated INR in plasma or blood |
| Platelet | TNX Curated Platelets |
| Hemoglobin | TNX Curated |
| Troponin I | TNX Curated Tropinin I Cardiac |
| Creatinine | TNX Curated Creatinine in serum |
| Hemoglobin A1c | TNX Curated Hemoglobin A1C in blood |
| Leukocytes | TNX Curated Leukocytes in Blood |
| Neutrophils | TNX Curated Neutrophils in Blood |
| Albumin | TNX Curated Albumin in serum |
| Low Density Lipoprotein | TNX Curated Cholesterol in LDL |
| High Density Lipoprotein | TNX Curated Cholesterol in HDL |
| Triglyceride | TNX Curated Triglycerides |

**Supplementary Table S2 Outcome Definition**

| **Outcomes** | **Definitions** |
| --- | --- |
| **Primary End-Point** |  |
| All-cause mortality, Acute Myocardial Infarction, and Cerebral Infarction | ICD-10-CM I21: Acute Myocardial Infarction  ICD-10-CM I63: Cerebral Infarction  TNX Curated: Deceased |
| **Secondary End-Point** |  |
| Bleeding Events | ICD-10-CM K92.0: Hematemesis  ICD-10-CM K92.1: Melena  ICD-10-CM K92.2: Gastrointestinal hemorrhage, unspecified  ICD-10-CM 160: Nontraumatic subarachnoid hemorrhage  ICD-10-CM I61: Nontraumatic intracerebral hemorrhage  ICD-10-CM I62: Other and Unspecified Nontraumatic Intracerebral Hemorrhage |
| All-cause mortality | TNX Curated: Deceased |
| Acute Myocardial Infarction | ICD-10-CM I21: Acute Myocardial Infarction |
| Cerebral Infarction | ICD-10-CM I63: Cerebral Infarction |
| Hospitalization | TNX Curated: Visit inpatient encounter  TNX Curated: Visit Inpatient Acute  TNX Curated: Visit Inpatient Non-acute  CPT 1013659 |
| Repeat Revascularization | SNOMED 415070008  SNOMED 868245005  SNOMED 868248007  SNOMED 868247002  SNOMED 868246006  SNOMED 868227005  SNOMED 11101003  SNOMED 232717009  SNOMED 232719007  SNOMED 232720001  SNOMED 232721002  SNOMED 232722009  SNOMED 232723004  SNOMED 232724005  ICD-10-PCS 02703ZZ  ICD-10-PCS 02713ZZ  ICD-10-PCS 02723ZZ  ICD-10-PCS 02733ZZ  ICD-10-PCS 02704ZZ  ICD-10-PCS 02714ZZ  ICD-10-PCS 02724ZZ  ICD-10-PCS 02734ZZ  ICD-10-PCS B212  ICD-10-PCS B213  CPT 92920  CPT 92921  CPT 1021163 |

**Supplementary Table S3. Sensitivity Analysis by Outcome and Follow-Up Time**

|  |  | No of Patients | | No of Events | |  |  |
| --- | --- | --- | --- | --- | --- | --- | --- |
| Outcomes | Follow Up Time | Prasugrel | Ticagrelor | Prasugrel | Ticagrelor | HR (95% CI) | p-value |
| Primary End-Points | 1 day - 1 year | 14895 | 14895 | 3073 (20.631%) | 4513 (30.299%) | 0.641 (0.613, 0.671) | <0.0001 |
|  | 1 week - 1 year | 14910 | 14910 | 2436 (16.338%) | 3652 (24.494%) | 0.627 (0.595, 0.66) | <0.0001 |
|  | 1 month - 1 year | 14910 | 14910 | 1895 (12.71%) | 2774 (18.605%) | 0.645 (0.608, 0.684) | <0.0001 |
|  | 3 Months - 1 year | 14910 | 14910 | 1442 (9.671%) | 2038 (13.669%) | 0.674 (0.63, 0.721) | <0.0001 |
|  | 1 day - 3 years | 14910 | 14910 | 3514 (23.568%) | 4913 (32.951%) | 0.665 (0.637, 0.695) | <0.0001 |
|  | 1 year - 5 years | 14910 | 14910 | 1717 (11.516%) | 2088 (14.004%) | 0.742 (0.696, 0.791) | <0.0001 |
|  | 1 day - 5 years | 14910 | 14910 | 3762 (25.231%) | 5180 (34.742%) | 0.667 (0.64, 0.696) | <0.0001 |
| Bleeding Events | 1 day - 1 year | 14895 | 14895 | 157 (1.054%) | 180 (1.208%) | 0.855 (0.69, 1.059) | 0.1514 |
|  | 1 week - 1 year | 14910 | 14910 | 149 (1%) | 156 (1.047%) | 0.936 (0.748, 1.172) | 0.5661 |
|  | 1 month - 1 year | 14910 | 14910 | 138 (0.926%) | 142 (0.952%) | 0.952 (0.753, 1.203) | 0.6791 |
|  | 3 Months - 1 year | 14910 | 14910 | 111 (0.744%) | 113 (0.758%) | 0.959 (0.738, 1.246) | 0.7543 |
|  | 1 day - 3 years | 14910 | 14910 | 332 (2.227%) | 317 (2.216%) | 1.003 (0.86, 1.17) | 0.9703 |
|  | 1 year - 5 years | 14910 | 14910 | 303 (2.032%) | 275 (1.844%) | 0.991 (0.841, 1.167) | 0.9116 |
|  | 1 day - 5 years | 14910 | 14910 | 431 (2.891% | 422 (2.83%) | 0.947 (0.828, 1.083) | 0.429 |
| All-cause Mortality | 1 day - 1 year | 14895 | 14895 | 331 (2.222%) | 587 (3.941%) | 0.557 (0.487, 0.637) | <0.0001 |
|  | 1 week - 1 year | 14910 | 14910 | 258 (1.73%) | 400 (2.683%) | 0.635 (0.543, 0.743) | <0.0001 |
|  | 1 month - 1 year | 14910 | 14910 | 191 (1.281%) | 227 (1.522%) | 0.824 (0.68, 0.999) | 0.0479 |
|  | 3 Months - 1 year | 14910 | 14910 | 156 (1.046%) | 159 (1.066%) | 0.958 (0.768, 1.195) | 0.7029 |
|  | 1 day - 3 years | 14910 | 14910 | 594 (3.984%) | 863 (5.788%) | 0.66 (0.6, 0.74) | <0.0001 |
|  | 1 year - 5 years | 14910 | 14910 | 450 (3.018%) | 470 (3.152%) | 0.853 (0.749, 0.97) | 0.0156 |
|  | 1 day - 5 years | 14910 | 14910 | 781 (5.238%) | 1063 (7.129%) | 0.689 (0.628, 0.756) | <0.0001 |
| Myocardial Infarction | 1 day - 1 year | 14895 | 14895 | 2688 (17.912%) | 3912 (26.264%) | 0.648 (0.616, 0.68) | <0.0001 |
|  | 1 week - 1 year | 14910 | 14910 | 2101 (14.091%) | 3193 (21.415%) | 0.622 (0.589, 0.658) | <0.0001 |
|  | 1 month - 1 year | 14910 | 14910 | 1632 (10.946%) | 2472 (16.579%) | 0.626 (0.588, 0.667) | <0.0001 |
|  | 3 Months - 1 year | 14910 | 14910 | 1226 (8.223%) | 1811 (12.146%) | 0.647 (0.602, 0.695) | <0.0001 |
|  | 1 day - 3 years | 14910 | 14910 | 2836 (19.021%) | 4079 (27.357%) | 0.656 (0.625, 0.688) | <0.0001 |
|  | 1 year - 5 years | 14910 | 14910 | 1139 (7.639%) | 1523 (10.215%) | 0.685 (0.634, 0.74) | <0.0001 |
|  | 1 day - 5 years | 14910 | 14910 | 2901 (19.457%) | 4162 (27.914%) | 0.654 (0.624, 0.686) | <0.0001 |
| Cerebral Infarction | 1 day - 1 year | 14895 | 14895 | 194 (1.302%) | 273 (1.833%) | 0.701 (0.583, 0.843) | 0.0001 |
|  | 1 week - 1 year | 14910 | 14910 | 170 (1.14%) | 238 (1.596%) | 0.704 (0.578, 0.857) | 0.0004 |
|  | 1 month - 1 year | 14910 | 14910 | 137 (0.919%) | 178 (1.194%) | 0.754 (0.604, 0.943) | 0.0128 |
|  | 3 Months - 1 year | 14910 | 14910 | 107 (0.718%) | 133 (0.892%) | 0.786 (0.609, 1.014) | 0.0632 |
|  | 1 day - 3 years | 14910 | 14910 | 314 (2.106%) | 405 (2.716%) | 0.749 (0.646, 0.868) | <0.0001 |
|  | 1 year - 5 years | 14910 | 14910 | 264 (1.771%) | 286 (1.918%) | 0.828 (0.701, 0.979) | 0.0272 |
|  | 1 day - 5 years | 14910 | 14910 | 402 (2.696%) | 497 (3.333%) | 0.757 (0.664, 0.864) | <0.0001 |
| Repeat Revascularization | 1 day - 1 year | 14895 | 14895 | 1355 (9.097%) | 762 (5.116%) | 1.791 (1.639, 1.957) | <0.0001 |
|  | 1 week - 1 year | 14910 | 14910 | 980 (6.573%) | 476 (3.192%) | 2.066 (1.851, 2.305) | <0.0001 |
|  | 1 month - 1 year | 14910 | 14910 | 786 (5.272%) | 368 (2.468%) | 2.129 (1.881, 2.41) | <0.0001 |
|  | 3 Months - 1 years | 14910 | 14910 | 409 (2.743%) | 213 (1.429%) | 1.892 (1.603, 2.233) | <0.0001 |
|  | 1 day - 3 years | 14910 | 14910 | 1631 (10.939%) | 975 (6.539%) | 1.68 (1.552, 1.818) | <0.0001 |
|  | 1 year - 5 years | 14910 | 14910 | 481 (3.226%) | 327 (2.193%) | 1.357 (1.179, 1.562) | <0.0001 |
|  | 1 day - 5 years | 14910 | 14910 | 1717 (11.516%) | 1051 (7.049%) | 1.63 (1.51, 1.76) | <0.0001 |

Purple: Ticagrelor, Green: Prasugrel

**Supplementary** **Figure S1. Distribution of Propensity Scores Before and After Matching**

**Supplementary Figure S2. Subgroup Analysis**. The figure presents hazard ratios (HRs) and 95% confidence intervals (CIs) for six clinical outcomes across multiple patient subgroups: (A) primary endpoints, (B) bleeding events, (C) all-cause mortality, (D) myocardial infarction, (E) cerebral infarction, and (F) repeat revascularization. Prasugrel demonstrated a consistent benefit in reducing primary endpoints, mortality, myocardial infarction, and cerebral infarction compared to ticagrelor across most subgroups, with HRs significantly less than 1. Bleeding risks were not significantly different overall, though some subgroup variations exist. Notably, prasugrel was associated with significantly higher rates of repeat revascularization in all analyzed subgroups (HRs > 1), indicating a potential trade-off despite its superior efficacy in ischemic outcomes.

**Supplementary Figure S3. Forest Plot for Sensitivity Analysis of Patient with Acute Coronary Syndrome.**

**Supplementary Figure S4. Forest Plot for Sensitivity Analysis of Patient with STEMI**

**Supplementary Figure S5. Forest Plot for Sensitivity Analysis of Patient with NSTEMI**
